# Event-Related Potentials Altered in Patients with Borderline Personality Disorder during Working Memory Tasks

**DOI:** 10.3389/fnbeh.2017.00067

**Published:** 2017-04-18

**Authors:** Ying Liu, Mingtian Zhong, Chang Xi, Xinhu Jin, Xiongzhao Zhu, Shuqiao Yao, Jinyao Yi

**Affiliations:** ^1^Medical Psychological Center, Second Xiangya Hospital, Central South UniversityChangsha, China; ^2^Center for Studies of Psychological Application, School of Psychology, South China Normal UniversityGuangzhou, China; ^3^Medical Psychological Institute, Central South UniversityChangsha, China

**Keywords:** borderline personality disorder, working memory, event-related potential, N-back task, workload

## Abstract

Whereas some studies have demonstrated impaired working memory (WM) among patients with borderline personality disorder (BPD), these findings have not been consistent. Furthermore, there is a lack of neurophysiological evidence about WM function in patients with BPD. The goal of this study was to examine WM function in patients with BPD by using event-related potentials (ERPs). An additional goal was to explore whether characteristics of BPD (i.e., impulsiveness and emotional instability) are associated with WM impairment. A modified version of the N-back task (0- and 2-back) was used to measure WM. ERPs were recorded in 22 BPD patients and 21 age-, handedness-, and sex-matched healthy controls (HCs) while they performed the WM task. The results revealed that there were no significant group differences for behavioral variables (reaction time and accuracy rate) or for latencies and amplitudes of P1 and N1 (all *p* > 0.05). BPD patients had lower P3 amplitudes and longer N2 latencies than HC, independent of WM load (low load: 0-back; high load: 2-back). Impulsiveness was not correlated with N2 latency or P3 amplitude, and no correlations were found between N2 latency or P3 amplitude and affect intensity scores in any WM load (all *p* > 0.05). In conclusion, the lower P3 amplitudes and longer N2 latencies in BPD patients suggested that they might have some dysfunction of neural activities in sub-processing in WM, while impulsiveness and negative affect might not have a close relationship with these deficits.

## Introduction

Borderline personality disorder (BPD) is a serious mental disorder that is characterized by a pervasive pattern of instabilities in affect regulation, impulse control, interpersonal relationships, and self-image (van Zutphen et al., 2015; Chanen and Thompson, 2016). Clinical theoreticians and researchers have proposed that the symptoms and behaviors of BPD are associated, at least in part, with disruptions in basic neurocognitive processes, and those neurocognitive impairments may moderate development of BPD (Judd, 2005; Fertuck et al., 2006). Neurocognitive deficits associated with BPD include dysfunctions in attention, concentration, memory and executive functions, such as impulse control, planning and problem solving (Judd, 2005). These dysfunctions might be for a faulty allocation of processing resources (Bazanis et al., 2002), which would be exemplified by a deficit in the efficacy of the central executive component of human working memory (WM; Oberauer et al., 2003).

WM, which supports online maintenance and manipulation of information (Baddeley, 2003), consists of three subcomponents (central executive, phonological storage and spatial information), and provides attention control over other cognitive abilities. But only a few studies have considered WM in BPD, and the results have been inconsistent. For example, whereas some studies found a WM deficit in BPD populations (O’Leary, 2000; Stevens et al., 2004; Lazzaretti et al., 2012), others did not (Sprock et al., 2000; Judd, 2005; Gvirts et al., 2012). When normal populations engage in a WM task, the parietal cortex and dorsolateral prefrontal cortex are activated (Curtis, 2006; Fang et al., 2016; Ng et al., 2016). The function of dorsolateral prefrontal cortex is abnormal in BPD patients (Lis et al., 2007; Rossi et al., 2015; Krause-Utz et al., 2016). Thus, WM deficits observed in BPD patients might implicate dysfunction in the dorsolateral prefrontal cortex or parietal cortex. However, there is insufficient direct neurophysiological evidence of WM impairment in BPD patients. Previous studies using accuracy rate and reaction time only generally explored whether WM deficits exist in BPD patients, without examining possible WM subcomponent deficits. Meanwhile, some of these studies didn’t eradicate the influence of medicine, comorbidity with other disorders, and could not conclude the real features of WM in BPD.

Event-related potentials (ERPs) measure electrical brain activity that is time-locked to sensory or cognitive events. ERPs elucidate the time course of ongoing brain activity during WM tasks and reflect the spatiotemporal sequence of cortical information processing (Kayser et al., 2006). ERPs are superior to behavioral or other neuroimaging measures, the latter of which have poor temporal resolution, when seeking information about the cognitive processing stages that contribute to WM abnormalities in BPD. Among the ERP components, N2 and P3 amplitudes have been reliably associated with WM function (Kim et al., 2014; Stroux et al., 2016). As a negative component was typical elicited between 200 ms and 350 ms poststimulus, N2 reflects retrieval of memory representations and perceptual comparisons (Patel and Azzam, 2005; Folstein and Van Petten, 2008). N2 is assumed to be an index of interference control (Donkers and van Boxtel, 2004; Folstein and Van Petten, 2008) and conflict monitoring/resolution. P3, a positive component typically elicited between 300 ms and 600 ms poststimulus, is associated with the general processes of attention control and stimulus categorization/evaluation (Bledowski et al., 2004; Rueda et al., 2004; Neuhaus et al., 2010a,b; Dai and Feng, 2011; Rossi et al., 2015).

Some studies have investigated N2 and P3 in subjects with BPD. For example, Houston et al. (2004) reported that adolescents who had BPD characteristics exhibited decrements in P3 amplitude. Ruchsow et al. (2008) recently found that BPD patients showed reduced P3 amplitude in a Go/Nogo task. However, there have been no studies investigating the N2/P3 characteristics in WM tasks in BPD patients. Several studies have focused on the performances of BPD patients in tasks with variable complexity or cognitive load. Stevens et al. (2004) reported that WM was impaired in BPD subjects, but WM function did not worsen when cognitive load was increased. Lazzaretti et al. (2012) only observed WM deficit in BPD patients when WM demands were high.

In light of this background, we recruited BPD patients and used the N2 and P3 components of ERPs to investigate the WM mechanism of BPD patients while performing an N-back task, while the effects medicine and comorbidity were controlled. Early sensory processing is the foundation of more complex cognition, and it influences WM performance (Tek et al., 2002). To examine whether there are early sensory defects in BPD, we recorded P1 and N1, which might be related to the early sensory stages of information processing (Thomas et al., 2013). Besides, impulsiveness and emotional instability are two core characteristics of BPD (Berlin et al., 2005; Domes et al., 2006; Jacob et al., 2010), and the degree of instability of these characteristics may be correlated with cognition impairment (Ruchsow et al., 2008; Svaldi et al., 2012; Hagenhoff et al., 2013). Therefore, we also examined whether impulsiveness and emotional instability influence WM performance of BPD patients.

## Materials and Methods

### Participants

Two professional psychiatrists diagnosed patients with BPD using Diagnostic and Statistical Manual of Mental Disorders-IV (*DSM-IV*) criteria (Maffei et al., 1997). Subjects were excluded from this study if they had schizophrenia, schizoaffective disorder, attention-deficit hyperactivity disorder (ADHD), delusional (paranoid) disorder, bipolar disorder, psychotic disorder, hypothyroidism, or seizure disorder, or any history of head injury, neurosurgery, or substance abuse. Patients with other Axis I/II disorders were also excluded. Patients were not taking any medication at the time of enrollment. The BPD group included 22 right-handed young subjects (14 males, 8 females; age: 22–27 years).

The control group comprised 21 age-, education- and handedness-matched healthy subjects (10 males, 11 females; age: 22–25 years). All subjects in the control group were interviewed by two professional psychiatrists to exclude *DSM-IV* criteria and other Axis I/II disorders. Criteria for inclusion in the control group were as follows: no current medical problems, no history of substance or alcohol abuse, and no history of psychiatric disorders among first-degree relatives.

All subjects had normal or corrected-to-normal vision. This study was carried out in accordance with the recommendations of “ethics committee of Central South University”. The protocol was approved by the ethics committee of  Central South University. Every subject signed an informed consent form in accordance with the Declaration of Helsinki. In the case of BPD patients, this consent form was also signed by a well-informed relative.

### Psychometric Instruments

#### Center for Epidemiological Studies Depression Scale (CES-D)

The CES-D was used to assess depressive levels of participants. This scale consists of 20 items, each of which is assigned a value from 1 to 4. Four items are positively worded and reverse-scored. The total score is computed by summing the 20 items, such that the range of scores is 20–80 (Natamba et al., 2014). The Chinese version of the CES-D has high internal consistency and construct validity (Xiao et al., 2016).

#### Barratt Impulsiveness Scale—11th Version (BIS-11)

The BIS-11 is one of the most widely used measures of impulsive personality traits. The 30 items are rated from 1 (rarely/never) to 4 (almost always/always). Items are summed to determine the overall impulsiveness score, with higher scores indicating greater impulsivity (Patton et al., 1995). The Chinese translation of the BIS-11 shows sufficient reliability and validity (Yao et al., 2007).

#### Childhood Trauma Questionnaire (CTQ)

The CTQ is a self-administered questionnaire that addresses childhood trauma in the following five areas: physical abuse, emotional abuse, sexual abuse, physical neglect and emotional neglect. The short form of this questionnaire includes 28 items that are scored on a five-point Likert scale ranging from 1 (never true) to 5 (very often true). There are five questions for each type of trauma, scored 5–25, with an additional three questions to assess minimization/denial (Lee et al., 2015). The Chinese version of the CTQ has good reliability and validity (Zhao et al., 2005).

#### Short Affect Intensity Scale (SAIS)

The SAIS has 20 items that are scored on a six-point Likert scale ranging from 1 to 6 (Geuens and De Pelsmacker, 2002). Three factors are analyzed with this scale: positive intensity (8 items), negative intensity (6 items), and serenity (6 items). There are no total scale scores. The score for each factor is a mean score of the items in that factor; consequently, the scores for individual factors range from 1 to 6. The Chinese version of the SAIS has good reliability and validity (Zhong et al., 2010).

### Stimulation and Task Procedures

**Figure 1:**
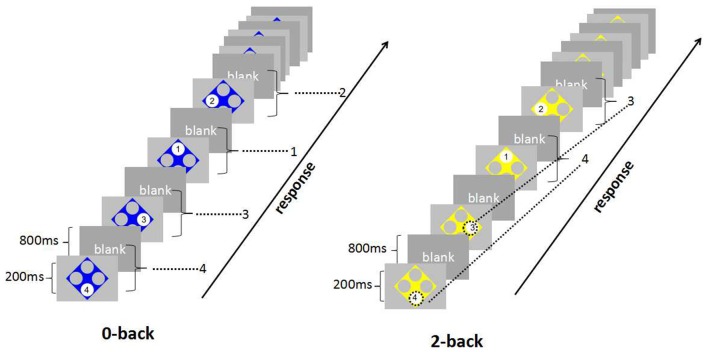
**The paradigm of *N*-back task**. For 0-back, the task required a simple button press in response to the number displayed. For 2-back, participants pressed the key corresponding to the number presented two trials before the current one.

A modified version of the N-back task was used to measure WM (Blokland et al., 2008; Baller et al., 2013). Participants performed a 0-back task and a 2-back task. As shown in Figure 1, numbers (1–4) of the N-back task were in a fixed position in one of four large white circles. Circles were positioned at each of the corners of a diamond-shaped square on a gray background of the screen. Stimuli were projected by using E-prime 2.0. Using their right index or middle finger, participants pressed one of four buttons to match the target stimulus. In this study, the 0-back task required a simple button press in response to the number displayed, while the 2-back task required participants to press the key corresponding to the number presented in two trials before the current one, therefore, the 2-back task requires on-line monitoring, updating and manipulation of remembered information and is assumed to place great demands on a number of key processes within WM (Glahn et al., 2005; Owen et al., 2005). Differing from traditional N-back task which judged if present number was similar to the n-back one, our task demanded participants to pressed one of four buttons to match the n-back one, which meant that the participants just had 25% chance to guess right, which was more difficult than traditional task.

There were 16 blocks and 8 blocks per condition. Each block consisted of 16 trials with a stimulus presentation time of 200 ms and interstimulus interval of 800 ms. There were 16-s rest intervals for subjects when they finished each block. The total experimental length was 8.2 min (492 s).

### Electrophysiological Recording and Analysis

As participants completed the N-back task, continuous EEG signals were acquired with a 64 channel electroencephalographeic system (EEG Nuamps, NeuroScan, Inc., El Paso, TX, USA). Electrodes were placed by using a 10/20 extended QuikCap system (NeuroScan, Inc., El Paso, TX, USA). References were placed at a vertex by default and off-line re-referenced to averaged mastoids. Impedanc values were kept lower than 5 KΩ for all electrodes. Horizontal electrooculograms (EOGs) were recorded with electrodes placed on the bilateral external canthi. Vertical EOGs were recorded from electrodes placed above and below the left eye. EEG data were sampled at 1000 Hz and analyzed offline with a 30-Hz low pass filter. Trials with undesired eye movements and eye blink artifacts were removed from analysis by a semiautomatic and manual block rejection procedure.

The continuous EEG was subsequently segmented beginning at 200 ms before stimulus onset and lasting for 800 ms. The baseline for ERP analysis was 100 ms before appearance of the target stimuli. Individual segments were excluded if the absolute voltage of each channel exceeded 100 μV. In each subject, artifact-free trials were averaged for each task (0-back/2-back) to obtain the corresponding ERP waves. Subjects with less than 30 epochs for each task were excluded. All analyses were performed by using Scan 4.3 and Curry 7.0 (NeuroScan, Inc., El Paso, TX, USA).

Three electrode positions (frontal: Fz; central: Cz; parietal: Pz) were chosen for statistical analyses of N2 and P3. Occipital electrodes (O1, Oz and O2) were selected for P1 and N1 because these components are usually maximal at these electrodes. ERP waves were analyzed in terms of peak latency and baseline-to-peak amplitude, as determined by visual inspection. Latency ranges for potentials were designated as follows: 50–150 ms for P1, 140–200 ms for N1, 200–400 ms for N2, and 250–500 ms for P3.

### Data Analysis

We used SPSS 17.0 to analyze all data. Demographic data of participants were analyzed by using a Chi-Square or *t*-test. Reaction time and accuracy data were analyzed by 2 (group: BPD vs. control) × 2 (WM load: 0-back vs. 2-back) repeated-measures ANOVA with WM load as a within-subject factor and group as a between-subject factor. ERP components (latency and amplitude) were analyzed by 2 (group: BPD vs. healthy control (HC)) × 2 (WM load: 0-back vs. 2-back) × 3 (electrodes: O1, Oz, O2/ Fz, Cz, Pz) repeated-measures ANOVA with WM load and electrodes as within-subject factors, and with group as a between-subject factor. Greenhouse–Geisser was used to correct compound symmetry violations in the ANOVAs. Correlations between psychological measures (impulsiveness and different affect intensity) and ERP components were calculated by Pearson’s correlation. Where appropriate, Cohen’s *d* and *η*^2^ were calculated as indices of effect size.

## Results

### Clinical Data

Clinical data are reported in Table 1. Higher scores for impulsiveness (*t* = 3.94, *p* < 0.001) and depression (*t* = 5.22, *p* < 0.001) were obtained for BPD patients compared to HCs. BPD patients had higher scores on CTQ subscales of emotional abuse (*t* = 2.20, *p* = 0.034), emotional neglect (*t* = 2.14, *p* = 0.040), and physical neglect (*t* = 3.68, *p* < 0.001), as well as on the SAIS subscale of negative intensity (*t* = 3.40, *p* < 0.001).

**Table 1:** **Clinical features**.

	BPD patients (*N* = 22)	HC (*N* = 21)	*t/χ*^2^	*p* value	Cohens’*d*
Age (years)	22.34 (1.04)	23.50 (0.74)	0.34	0.736	–
Sex (male/female)	14/8	10/11	1.87	0.172	–
CES-D	38.95 (8.95)	26.84 (4.31)	5.22	<0.001	1.72
BIS	65.73 (7.79)	57.43 (5.84)	3.94	<0.001	1.21
**CTQ**
Emotional abuse	7.23 (2.69)	5.81 (1.24)	2.20	0.034	0.68
Physical abuse	5.86 (1.61)	5.34 (0.56)	1.43	0.166	–
Sexual abuse	5.36 (1.05)	5.05 (0.44)	1.29	0.208	–
Emotional neglect	9.82 (4.62)	7.39 (2.57)	2.14	0.040	0.65
Physical neglect	8.55 (2.99)	5.83 (1.72)	3.68	<0.001	1.12
**SAIS**
Positive intensity	4.25 (0.88)	3.89 (0.61)	1.56	0.128	–
Negative intensity	4.31 (0.88)	3.44 (0.55)	3.40	<0.001	1.19
Serenity	3.11 (0.79)	2.76 (0.69)	1.52	0.136	–

*Age, CES-D, BIS, CTQ and SAIS express as mean (SD); CES-D, The Center for Epidemiological Studies Depression Scale; BIS, The Barratt Impulsiveness Scale11th version; CTQ, The Childhood Trauma Questionnaire-Short Form; SAIS, The Short affect intensity scale*.

### Behavioral Results

Mean reaction time and accuracy data for both groups under each condition are reported in Table 2. On mean reaction time, there were no significant group differences (*F*_(1,41)_ = 0.001, *p* = 0.979), no significant effects of WM load (*F*_(1,41)_ = 0.79, *p* = 0.380), and no significant Group × WM load interaction effects (*F*_(1,41)_ = 0.22, *p* = 0.644). However, WM load had a significant effect on accuracy (*F*_(1,41)_ = 312.38, *p* < 0.001, *η*^2^ = 0.88). Low WM load had a higher accuracy rate (97.7% ± 3.5%) than high WM load (60.9% ± 12.3%), but no significant group differences (*F*_(1,41)_ = 0.21, *p* = 0.652) or significant main Group × WM load interactions (*F*_(1,41)_ = 0.04, *p* = 0.848) were observed on accuracy.

**Table 2:** **Reaction time [ms] and performances [%] for *N*-back tasks**.

	BPD patients (*N* = 22)		HC (*N* = 21)	
	0-back	2-back	0-back	2-back
Reaction time	369.79 (46.88)	375.69 (85.24)	362.90 (43.50)	381.83 (75.70)
Performances	97.09 (4.78)	60.69 (10.54)	98.33 (1.11)	61.12 (14.08)

*Reaction time and Performances express as mean (SD)*.

### ERP Components

**Figure 2:**
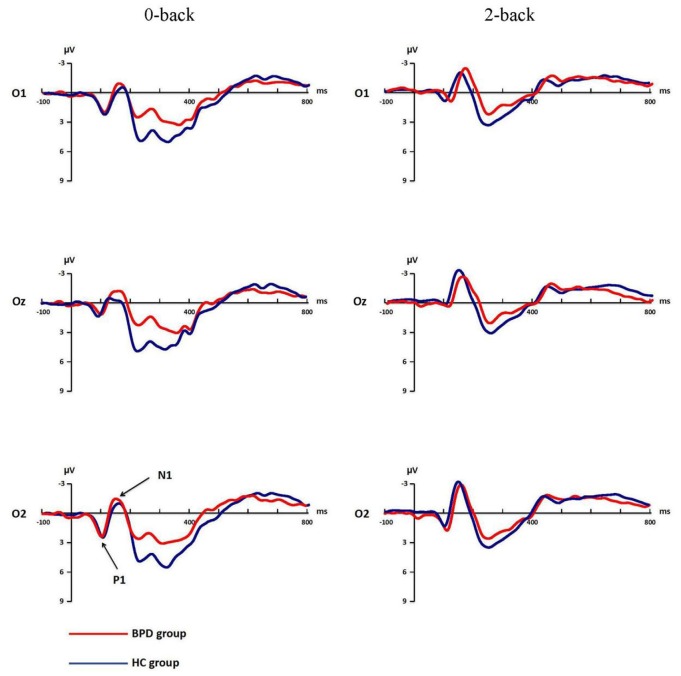
**Grand-average event-related potential (ERP) waveforms of P1, N1 for 0-back and 2-back in borderline personality disorder (BPD) and healthy control (HC) groups**. The electrodes of O1, Oz and O2 were chosen to record P1 and N1. P1 and N1 changed under 0-back and 2-back in both groups.

**Figure 3:**
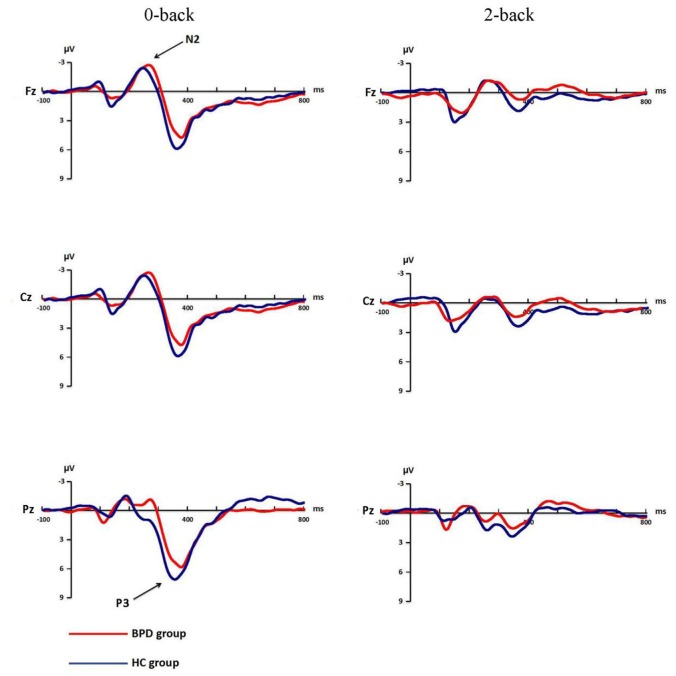
**Grand-average ERP waveforms of N2, P3 for 0-back and 2-back in BPD and HC groups**. The electrodes of Fz, Cz and Pz were chosen to record N2 and P3. N2 and P3 changed under 0-back and 2-back in both groups.

Mean amplitudes and latencies of P1, N1, N2 and P3 for each WM load are shown for both groups in Table 3. P1 and N1 grand-average ERPs are shown in Figure 2. N2 and P3 grand-average ERPs are shown in Figure 3.

**Table 3:** **Mean ERP amplitude and latency for *N*-back tasks by group**.

		BPD patients (*N* = 22)		HC (*N* = 21)	
		0-back	2-back	0-back	2-back
Amplitude	P1	3.76 (2.78)	2.86 (2.48)	4.55 (2.97)	2.48 (2.02)
	N1	−3.35 (3.67)	−5.34 (3.61)	−2.97 (4.21)	−5.12 (4.62)
	N2	−5.18 (3.07)	−2.75 (2.64)	−4.83 (4.07)	−2.00 (2.72)
	P3	7.36 (3.59)	2.70 (2.51)	9.74 (5.05)	4.74 (2.81)
Latency	P1	106.58 (14.11)	108.92 (16.63)	107.51 (17.30)	104.54 (16.78)
	N1	155.05 (17.46)	158.29 (14.05)	148.92 (20.01)	151.76 (17.55)
	N2	253.24 (18.62)	261.44 (29.31)	240.21 (24.67)	249.11 (25.47)
	P3	377.35 (25.94)	363.24 (26.63)	358.75 (22.67)	361.00 (32.22)

*P1, N1, N2, P3 amplitude and Latency express as mean (SD)*.

#### P1 Amplitude

The main effect of WM load in P1 amplitude was significant (*F*_(1,41)_ = 18.38, *p* < 0.001, *η*^2^ = 0.31). P1 amplitude was more positive under low WM load (4.16 ± 3.15 μV) compared to high WM load (2.67 ± 2.56 μV). The effect of electrodes was also significant (*F*_(2,82)_ = 14.06, *p* < 0.001, *η*^2^ = 0.26), the amplitude of Oz was the minimum (2.61 ± 2.49 μV). The group difference in P1 amplitude, interactions of WM load × Electrodes, WM load × Group, Electrodes × Group, and WM load × Group × Electrodes were not significant (all *F* < 2.60, *p* > 0.08).

#### P1 Latency

The repeated-measures ANOVA on P1 latency showed significant main effect of electrodes (*F*_(2,41)_ = 10.52, *p* < 0.001, *η*^2^ = 0.20) and interaction of WM load × Electrodes (*F*_(2,41)_ = 8.58, *p* < 0.001, *η*^2^ = 0.17). Further analysis revealed that O2 had a shorter latency (102.20 ± 14.25 ms) compared to O1 (110.06 ± 16.47 ms, *p* < 0.001, Cohen’s *d* = 0.51) or Oz (108.87 ± 21.19 ms, *p* = 0.017, Cohen’s *d* = 0.37) under low WM load. Oz had a shorter latency (102.31 ± 20.22 ms) compared to O1 (110.40 ± 17.33 ms, *p* < 0.001, Cohen’s *d* = 0.43) or O2 (106.48 ± 16.34 ms, *p* = 0.016, Cohen’s *d* = 0.23) under high WM load. The group difference, main effect of WM load and remaining interactions were not significant (all *F* < 1.09, *p* > 0.336).

#### N1 Amplitude

The ANOVA conducted on N1 amplitude revealed significant main effects of WM load (*F*_(1,41)_ = 27.03, *p* < 0.001, *η*^2^ = 0.40), electrodes (*F*_(2,82)_ = 12.18, *p* < 0.001, *η*^2^ = 0.23). The interaction of WM load × Electrodes was also significant (*F*_(2,82)_ = 5.47, *p* = 0.011, *η*^2^ = 0.12), *post hoc* analysis showed that the N1 amplitude was smallest in O1 under both low (−2.58 ± 3.47 μV) and high (−4.19 ± 3.39 μV) WM load. The group difference and the remaining interactions were not significant (all *F* < 2.01, *p* > 0.152).

#### N1 Latency

Electrodes exhibited a main effect on N1 latency (*F*_(2,82)_ = 4.66, *p* = 0.015, *η*^2^ = 0.10). The N1 latency of Oz (149.26 ± 23.31 ms) was shorter than the N1 latency of O1 (156.39 ± 20.41 ms, *p* = 0.001, Cohen’s *d* = 0.33) or O2 (154.86 ± 17.36 ms, *p* = 0.040, Cohen’s *d* = 0.27), but there was no significant difference between O1 and O2 (*p* = 0.572). None of the group difference, main effect of WM load and remaining interactions were significant (all *F* < 1.80, *p* > 0.188).

#### N2 Amplitude

There were significant main effects of WM load (*F*_(1,41)_ = 30.54, *p* < 0.001, *η*^2^ = 0.43) and electrodes (*F*_(2,82)_ = 10.47, *p* = 0.001, *η*^2^ = 0.20). The interaction of WM load × Electrodes (*F*_(2,82)_ = 12.12, *p* < 0.001, *η*^2^ = 0.23) was also significant, N2 amplitude was smaller for Pz (−3.35 ± 4.35 μV) than for Fz (−5.83 ± 3.60 μV, *p* < 0.001, Cohen’s *d* = 0.62) or Cz (−5.85 ± 4.38 μV, *p* = 0.001, Cohen’s *d* = 0.57) under low load, while the amplitude of Fz (−3.13 ± 2.77 μV) was larger than that of Pz (−1.78 ± 3.34 μV, *p* = 0.010, Cohen’s *d* = 0.44) or Cz (−2.22 ± 3.09 μV, *p* < 0.001, Cohen’s *d* = 0.31) under high WM load. The group difference and other interactions were not significant (all *F* < 2.02, *p* > 0.151).

#### N2 Latency

A significant group difference was found in N2 latency (*F*_(1,41)_ = 4.33, *p* = 0.044, *η*^2^ = 0.10). BPD patients had longer latencies (257.34 ± 20.03 ms) than HC (244.66 ± 19.91 ms). The main effect of electrodes (*F*_(2,82)_ = 23.97, *p* < 0.001, *η*^2^ = 0.37) and the Group × Electrode interaction (*F*_(2,82)_ = 3.95, *p* = 0.034, *η*^2^ = 0.09) were also significant, indicating that BPD patients had a longer latency (249.64 ± 35.28 ms) than HC (226.21 ± 33.47 ms, *p* = 0.011) in the Pz electrode only. The main effect of WM load and other interactions were not significant (all *F* < 3.63, *p* > 0.064).

#### P3 Amplitude

Significant group difference was found in P3 amplitude (*F*_(1,41)_ = 5.62, *p* = 0.023, *η*^2^ = 0.12), BPD patients had a smaller P3 amplitude (5.03 ± 2.61 μV) compared to controls (7.24 ± 3.46 μV). WM load (*F*_(1,41)_ = 66.46, *p* < 0.001, *η*^2^ = 0.62), electrodes (*F*_(2,82)_ = 41.71, *p* < 0.001, *η*^2^ = 0.50) and WM load × Electrodes interaction (*F*_(2,82)_ = 20.68, *p* < 0.001, *η*^2^ = 0.33) all had significant effects. *Post hoc* analysis showed that P3 amplitude was significant larger for Pz (10.92 ± 5.18 μV) compared to Fz (5.73 ± 4.24 μV, *p* < 0.001, Cohen’s *d* = 1.10) or Cz (8.99 ± 5.36 μV, *p* < 0.001, Cohen’s *d* = 0.37) under low WM load, the P3 amplitude for Fz (2.85 ± 3.19 μV) was smaller than for Cz (3.96 ± 3.12 μV, *p* = 0.003, Cohen’s *d* = 0.35) or Pz (4.34 ± 3.04 μV, *p* = 0.0311, Cohen’s *d* = 0.48) under high WM load. The remaining interactions were not significant (all *F* < 1.36, *p* > 0.258).

#### P3 Latency

Electrodes significantly affected P3 latency (*F*_(2,82)_ = 6.98, *p* = 0.004, *η*^2^ = 0.15). Fz had a longer latency (372.37 ± 27.46 ms) than Cz (365.03 ± 22.76 ms, *p* = 0.030, Cohen’s *d* = 0.29) or Pz (357.85 ± 24.51 ms, *p* = 0.005, Cohen’s *d* = 0.56). Furthermore, Cz had a longer latency than Pz (*p* = 0.033). There was no significant group difference or main effect of WM load, as well as no significant interactions for WM load × Group, Electrodes × Group, WM load × Electrodes, or WM load × Electrodes × Group (all *F* < 3.02, *p* > 0.09).

### Correlations among N2 Latency, P3 Amplitude and Psychological Measures

Pearson’s correlation analyses showed that N2 latency was not significantly correlated with P3 amplitude in the 0-back task (*r* = −0.05, *p* = 0.731) or 2-back task (*r* = −0.25, *p* = 0.100).

Pearson’s correlation analyses showed that impulsiveness, as detected by the BIS, was not correlated with N2 latency under any WM load. There were no significant correlations between N2 latency and affect intensity scores, or between P3 amplitude and impulsiveness or affect intensity scores (all *p* > 0.05).

## Discussion

To the best of our knowledge, this study is the first investigation of WM in BPD by ERPs. Our behavioral results showed no differences between BPD patients and HCs in N-back task. Although there were no significant group differences in accuracy or mean reaction time, the BPD group showed lower P3 amplitude and longer N2 latency results compared to the control group. As expected, we found that the accuracy and the P1, N2 and P3 amplitudes decreased as WM load increased. Nevertheless, the reduced P3 amplitude and longer N2 latency in the BPD group were independent of WM load. Meanwhile, in this study, neither impulsiveness nor negative affect was the main factor lead to the deficits of WM in BPD.

Despite the lack of general agreement about its functional role, N2 has been correlated with ease of visual information encoding (Nittono et al., 2007). N2 latency has been used as a physiological marker of the timing of access to different properties of a stimulus (Folstein and Van Petten, 2008). The finding that N2 latency was longer in BPD patients than in HCs suggests that stimulus analysis and evaluation during information encoding in WM might be slower in BPD patients compared to healthy individuals.

BPD patients showed lower P3 amplitudes than HCs. P3 amplitude in the WM task is related to the allocation of attention necessary for WM functioning. Kok (2001) reported a relationship between P3 amplitude and attentional resource allocation. Linden (2005) found P3 to be related to brain regions involved in attention, such as the parietal lobe, temporo-parietal junction, lateral prefrontal areas, and cingulate gyrus. Studies about ADHD (Kim et al., 2014; Stroux et al., 2016), which is often comorbid with BPD (Speranza et al., 2011), concluded that a diminished P3 amplitude could be interpreted as an inefficient allocation of attention in WM. These observations suggest that there is abnormal neural activity on allocating attention resources in BPD patients.

The obvious WM load effect in our study proved the effectiveness of the N-back task. However, the reduced P3 amplitude and longer N2 latency results were independent of WM load, consistent with a previous report that WM is impaired in BPD subjects regardless of WM load (Stevens et al., 2004). The N2 latency WM load-independence might occur because the speed of processing for a 0-back task is not different from that of a 2-back task. For P3 amplitude, consistent with other studies (Posner et al., 2002; Stevens et al., 2004; Lazzaretti et al., 2012), we found no correlation between P3 amplitude and affect intensity or impulsiveness suggested that the diminished P3 amplitude was not modulated by affect intensity and impulsiveness. Previous studies found that the information processing stages are not parallel, and those impairments in one stage may affect other stages (Di Russo et al., 2010; Portella et al., 2012). We did not observe any correlation between N2 latency and P3 amplitude, which suggests that the attenuated P3 amplitude might not be related to the compensation of the longer N2 latency. Based on these findings, we speculate that there was a general dysfunctional attention allocation in WM rather than specific problems due to increased demand on the WM load in BPD patients.

Hagenhoff et al. (2013) reported that perceptual processing and response selection are unaffected in BPD. Similarly to that report, our findings suggested that there were no group differences in P1 and N1. Both P1 and N1 are associated with early, rapid processing of stimuli, encoding, attentional focus and discrimination. These results suggest that BPD patients might not have deficits in the early phase of WM.

No differences of behavioral results were found between BPD group and HC group, which were inconsistent with our ERPs results. These inconsistent findings between behavioral data and imaging data were also found in many previous imaging studies (Karayanidis et al., 2000; Ruchsow et al., 2008; Myatchin et al., 2009), which suggested that EPRs results might be more sensitive, efficient and convinced than behavioral data.

The results of lower P3 amplitudes and longer N2 latencies in BPD patients revealed that BPD patients might have some abnormal brain activities in sub-processing of WM, especially in the process of allocating attention resources and the speed of stimulus analysis and evaluation during information encoding, furthermore, these abnormalities were independent of WM load. Although these detriments might not always be observed in behavioral performance, these findings could also provide some theoretical supports for the dysfunction of WM or cognition in patients with BPD. Even no significant correlations were found between ERPs indexes (N2 latency and P3 amplitude) and impulsiveness or negative affect intensity, we still could not conclude that the abnormalities of ERPs during WM tasks are independent of the symptoms of BPD, just as Fertuck et al. (2006) concluded that cognitive impairment is a key moderator in the development of BPD, influences the formation of insecure disorganized attachment and dissociation and interferes with cognitive development in the interpersonal arena. In the future, more attention should be paid to the relationship between impairment of WM and symptoms of BPD, and the WM and other cognition function should be considered in the diagnosis and the development of BPD. Fertuck et al. (2006) has found that BPD patients with higher executive control and higher performance on visual memory tasks were more likely to finish treatment, in this study, we also found that BPD patients had some dysfunctions of neural activity when finishing WM tasks, which suggested that BPD patients might be hard to allocate more attention resources effectively on the process of treatment, therefore, WM training and other cognitive training might have some advantages in improving the effectiveness of treatment in BPD.

This study has several limitations. First, although there is high temporal resolution, the poor spatial resolution of ERPs makes it difficult to identify which brain regions are related to the impairment of WM in BPD patients. Second, no patients had taken medicine in this cross-sectional study. Therefore, it is unclear whether medicine or psychological treatments would reduce impairment of WM. Further studies tracking the performance of BPD patients after taking medicine would help us to identify which impairment of WM are related to symptoms of BPD. Third, previous research suggests there are sex differences in BPD (Hoertel et al., 2014), whereas we did not analyze differences of WM between males and females with BPD. Therefore, sex differences of WM in BPD should be considered in the future. Finally, although no correlation was found between negative emotion and BPD performance in WM, an emotional N-back task should be used to explore the relationship between impaired WM and emotional dysfunction directly, since instability in affect regulation is a core symptom in BPD patients.

## Author Contributions

YL was responsible for analyzing data and writing the manuscript. MZ designed the experiment and wrote the manuscript with YL. CX, XJ, XZ and SY were responsible for data collection. JY was responsible for designing the study and revising the manuscript.

## Conflict of Interest Statement

The authors declare that the research was conducted in the absence of any commercial or financial relationships that could be construed as a potential conflict of interest.
